# The Effect of Thyme Essential Oil on Liver Injuries Caused by Renal Ischemia-Reperfusion in Rats

**DOI:** 10.1155/2022/2988334

**Published:** 2022-10-26

**Authors:** Reza Rostami, Zahra Eslamifar, Sedighe Nazemi, Seyedeh Zeinab Hosseini, Mohammad mehdi Behvandi, Leila Jafaripour

**Affiliations:** ^1^Razi Herbal Medicines Research Center, Lorestan University of Medical Sciences, Khorramabad, Iran; ^2^Department of Medical Laboratory Sciences, School of Paramedical Sciences, Dezful University of Medical Sciences, Dezful, Iran; ^3^Department of Internal Medicine, School of Medicine, Dezful University of Medical Sciences, Dezful, Iran; ^4^Hepatitis Research Center, Lorestan University of Medical Sciences, Khorramabad, Iran; ^5^Student Research Committee, Dezful University of Medical Sciences, Dezful, Iran; ^6^Department of Anatomy, School of Medicine, Dezful University of Medical Sciences, Dezful, Iran

## Abstract

Liver damage occurs following renal ischemia-reperfusion (RIR) that can cause inflammation and inflammatory cytokines activated after kidney injury. In this study, thyme essential oil (TE) with antioxidant and anti-inflammatory properties was used to reduce liver damage induced by renal IR. 32 male rats were randomly divided into 4 equal groups: (1) control, (2) RIR, (3) RIR+TE, and (4) TE. Rats received TE as a pretreatment at a dose of 0.5 ml/kg for one week. Then, under anesthesia for 45 minutes for ischemia, the kidneys of the animals were closed with clamps, and reperfusion was performed for 24 hours. Animal serum was isolated to evaluate alkaline phosphatase (ALP), aspartate aminotransferase (AST), and alanine aminotransferase (ALT) parameters. The liver of rats was examined for the measurement of malondialdehyde (MDA), nitric oxide (NO), glutathione (GSH), glutathione peroxidase (GPX), catalase (CAT), and expression of genes such as interleukin-6 (IL-6), tumor necrosis factor-*α* (TNF-*α*), and caspase-3. ALP, AST, ALT, MDA, NO, IL-6, TNF-*α*, and caspase-3 increased significantly in the RIR group compared to the control group (*p* < 0.05). GSH, GPX, and CAT decreased significantly in the RIR group compared to the control group (*p* < 0.05). TE caused a decrease in ALP, AST, ALT, MDA, NO, IL-6, and TNF-*α* compared to the RIR group and caused an increase in the amount of GSH, GPX, and CAT in the RIR group (*p* < 0.05). This study showed that TE has antioxidant and anti-inflammatory properties that reduce liver damage induced by RIR.

## 1. Introduction

Acute kidney injury (AKI) is one of the most common problems of patients admitted to hospital intensive care units. The mortality rate is 11%, and if it is associated with dysfunction of other organs, it increases to 45-60% [1]. Renal ischemia reperfusion (RIR) is one of the causes of AKI that occurs following kidney transplantation [2]. RIR may cause dysfunction in other organs such as the liver, brain, lungs, and heart. RIR causes the accumulation of reactive oxygen species (ROS), followed by oxidative stress [2]. Possibly, oxidative stress, systemic release of inflammation, and cytokines are involved in injuries to other organs [3]. Following RIR and kidney transplantation, inflammatory cytokines are released and activated from the damaged kidney which can cause damage to the liver tissue [4]. RIR causes liver damage by decreasing hepatic glutathione (GSH) and antioxidant enzymes such as catalase (CAT) and glutathione peroxidase (GPX) and increasing malondialdehyde (MDA) levels, increasing levels of inflammatory cytokines such as interleukin 6 (IL-6) and tumor necrosis factor *α* (TNF-*α*) [5–7]. Also, serum levels of liver enzymes such as aspartate aminotransferase (AST), alkaline phosphatase (ALP), and gamma-glutamyltransferase (GGT) increase during RIR [7]. Due to the sensitivity of liver function, if it does not regenerate quickly, dysfunction of this organ will occur [4].

Thymus vulgaris Leaf is an aromatic plant that grows mainly in the Mediterranean region. Thyme essential oil (TE) consists of thymol, carvacrol, eugenol, saponins, steroids, alkaloids, flavonoids, polyunsaturated fatty acids, cinnamon, paracetamol, and vitamins A and C [8–10]. The phenolic component of thyme oil has strong antimicrobial activity, and its antioxidant activity strengthens the immune system and disease resistance [8].

The aim of our study was to investigate the effect of thyme oil on oxidative stress, apoptosis, and inflammatory of the liver following RIR. In this study, parameters such as AST, alanine aminotransferase (ALT), ALP, oxidative stress tests, liver antioxidant enzymes, expression of caspase-3, TNF-*α*, and IL-6 were investigated.

## 2. Materials and Methods

### 2.1. Preparation of Thyme Oil Essential

Zataria multiflora Boiss (Shirazi thyme leaves) was purchased from Shiraz markets and powdered by grinding machine after drying. 100 gr of thyme powder along with 500 ml distilled water was poured into an Erlenmeyer flask and heated at 100°C for 2 hours. After that, its essential oil was extracted through steam distillation by a clevenger device (brand: BORO G). This device has a section known as condenser which converts steam and essential molecules into liquid while passing through it. This liquid, essential oil, is then stored in the containers away from light at 4°C in the refrigerator until injection.

### 2.2. Animals

In this study, 32 male Wistar rats weighing approximately 200-220 gr were kept in the animal house of Dezful University of Medical Sciences in the same conditions, with a temperature of 22-24°C and a humidity of 45-55% with 12 hours of darkness and light. Throughout the treatment protocol, water and food were freely available to the animals. This research was performed with the approval and based on the guidelines of Animal Care and Ethics Committee of Dezful University of Medical Sciences, Dezful, Iran (ethical number: IR.DUMS.REC.1395.5).

### 2.3. Experimental Design

Thirty-two male Wistar rats were randomly divided into four groups of eight: group 1—control, under anesthesia, laparotomy without renal ischemia-reperfusion surgery; group 2—ischemia reperfusion (RIR), 45 minutes of ischemia and then 24 hours of perfusion; group 3—RIR + TE, RIR group with injection of 0.5 ml/kg of TE intraperitoneally [11, 12] for 1 week as a pretreatment, a week before RIR surgery; and group 4—TE, receiving 0.5 ml/kg of TE intraperitoneally for 1 week.

### 2.4. Renal Ischemia Reperfusion Surgery

Animals were not allowed to eat something by mouth, and they were kept NPO for 8 hours before surgery. Rats were anesthetized with 75 mg/kg ketamine and 8 mg/kg xylazine. A midline incision was made in the abdomen, the kidney pedicle was closed for 45 minutes, and then reperfusion was performed for 24 hours. Blood was taken from the hearts of animals under anesthesia, and their livers were isolated for biochemical analysis and gene expression.

### 2.5. Measurement of BMI, Liver Weight, and Daily Water and Food Intake

The body weight in rats was measured at the beginning of treatment and before death. Body mass index (BMI) was calculated through this formula: BMI = body weight (grams)/length^2^ (cm^2^) [13]. Also, the weight of the livers was measured.

The daily water and food consumption of each cage was measured for one week, and the average water and food intake was calculated [13].

### 2.6. Measurement of Serum Parameters

The blood sample was centrifuged to separate serum at 3000 rpm for 10 minutes. ALP, AST, and ALT in the serum were measured with Pars Azmon commercial kits by autoanalyzer tool.

### 2.7. Measurement of Biochemical Parameters

#### 2.7.1. Measurement of Malondialdehyde (MDA)

Liver MDA was performed by thiobarbituric acid method [14]. Briefly, 25 *μ*l of sample and 500 *μ*l of 2 M AcONa buffer (pH = 3.5) with 0.2% TBA were mixed and incubated for 1 h at 95°C. Then, 500 *μ*l of 50 mM KH2PO4 buffer (pH = 6.8) was added to each sample and centrifuged for 5 minutes at 4°C; and finally, absorption of the supernatant was read by spectrophotometry at 532 *μ*m.

#### 2.7.2. Measurement of Nitric Oxide (NO)

Liver nitric oxide was measured by Griess method. In summary, a mixture of 50 *μ*l of 1% sulfonamide (in 5% H_3_PO_4_) and 50 *μ*l of a solution of 0.1% naphthylethylenediamine and 100 *μ*l of samples was prepared; then, its absorption at 560 nm was read by the ELISA reader instrument [15].

#### 2.7.3. Measurement of Catalase (CAT)

Liver catalase was performed by the Aebi method [16]. Briefly, 0.5 ml H_2_O_2_ (75 mM) was added to 1.5 ml of 0.1 M phosphate buffer (pH = 7) and 50 *μ*l of sample in the reaction mixture. Its absorption reduction was read at 240 nm for 1 minute with a spectrophotometer instrument.

#### 2.7.4. Measurement of Glutathione Peroxidase (GPX)

Liver GPX was performed by a fielding method [17]. In summary, the reaction mixture consisted of 25 *μ*l of a homogenized liver sample with 1.25 ml of reagent (4 mmol/l glutathione, 0.5 mmol/l glutathione reductase, and 0.034 mmol/l NADPH), 100 *μ*l cumene hydroperoxide (0.18 mmol) was prepared, and the reduction of NADPH adsorption at 470 nm was read by ELISA reader instrument.

#### 2.7.5. Measurement of Glutathione (GSH)

Liver GSH was performed by Zhang and Kirkham method [18]. Briefly, 0.2 mM NADPH, 100 mM phosphate buffer (pH = 7.5), 5 mM EDTA, 0.6 DTNB, and 3 GR units were added to this 0.1 ml sample reaction mixture. And its absorption at 412 nm was read by a spectrophotometer instrument.

### 2.8. Real-Time Quantitative PCR for Inflammatory Cytokines and Apoptosis Genes

Using Trizol reagent, RNA of all liver samples was extracted according to the manufacturer's instructions (AnCell Iran). The concentration and purity of RNA were determined by Thermo NanoDrop. Using the CDNA synthesis kit (AnCell, Iran), the complementary DNA of the first strand was synthesized according to the manufacturer's instructions. Expression of TNF-*α*, interleukin-6, and caspase-3 in the liver was detected using RT-qPCR. Each 20 *μ*l reaction consisted of 1 *μ*l of cDNA, 10 *μ*l SYBR Green qPCR Mix (1 X), 1 *μ*l of primer, and 8 *μ*l of DEPC water. The reactions were subjected to an initial denaturation of 95°C for 10 minutes before thawing, then 40 cycles at 95°C for 10 seconds, and annealing for 30 seconds at 60°C, and then final extension step at 72°C for 10 seconds. Calculation of gene-specific efficiencies and normalization to the mean expression of *β*-actin were calculated [19]. Used primer sequences has been mentioned in Table 1.

### 2.9. Statistical Analysis

Data were analyzed by SPSS 23 software and one-way analysis of variance (ANOVA). The significance level of the data was considered *p* value < 0.05. Value was expressed as mean ± standard error of the mean (SEM). The diagrams were drawn with GraphPad Prism software.

## 3. Result

### 3.1. The Effect of TE on Initial Body Weight, Final Body Weight, Liver Weight, BMI, and Daily Food and Water Intake

In the present study, there was no significant difference in initial body weight between different groups (*F* (3, 28) = 0.46, *p* = 0.71). One-way ANOVA showed no significant difference between the groups in final body weight (*F* (3, 28) = 0.58, *p* = 0.63) (Table 2).

In this study, a significant statistical difference in liver weight was observed between different groups (*F* (3, 28) = 4.78, *p* = 0.008). Liver weight in the RIR group showed a significant increase compared to the control group (*p* < 0.05). Liver weight in the RIR+TE and TE groups showed a significant decrease compared to the RIR group (*p* < 0.05). No significant difference was found between sham and TE groups in liver weight (*p* > 0.05) (Table 2).

There was no significant difference in BMI between different groups (*F* (3, 28) = 0.26, *p* = 0.85). No significant difference in food intake was observed between different groups (*F* (3, 28) = 0.33, *p* = 0.8). No significant statistical difference was observed between groups in water intake (*F* (3, 28) = 0.15, *p* = 0.91) (Table 2).

### 3.2. The Effect of TE on Liver Function following RIR

**Figure 1:**
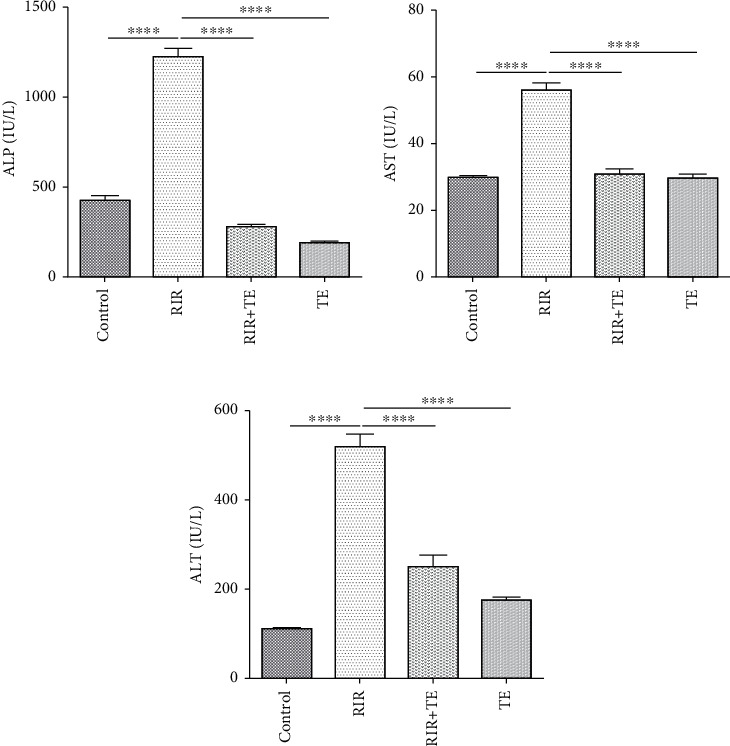
Effect of TE on liver function test following RIR. Data are expressed as mean + standard error mean (SEM). Values of this study were analyzed using one way ANOVA. Tukey test was used to compare the two groups. ^∗∗∗∗^ shows a significant difference between the two groups with *p* < 0.0001. ^∗∗∗^ shows a significant difference between the two groups with *p* < 0.001. ^∗∗^ shows a significant difference between the two groups with *p* < 0.01. ^∗^ shows a significant difference between the two groups with *p* < 0.05.

A significant difference in serum ALP level was observed between different groups (*F* (3, 28) = 274.97, *p* < 0.0001). Serum ALP in the RIR group showed a significant increase compared to the control group (*p* < 0.0001). Serum ALP in the RIR+TE and TE groups showed a significant decrease compared to the RIR group (*p* < 0.0001). In this study, no significant difference was found between sham and TE groups in serum ALP (*p* > 0.05) (Figure 1(a)).

In this study, one-way ANOVA showed a significant difference between the groups in serum AST (*F* (3, 28) = 69.84, *p* < 0.0001). Serum AST in the RIR group showed a significant increase compared to the control group (*p* < 0.0001). Serum AST in the RIR+TE and TE groups showed a significant decrease compared to the RIR group (*p* < 0.0001). In this study, no significant difference was found between the control and TE groups in serum AST (*p* > 0.05) (Figure 1(b)).

In the present study, there was a significant difference in serum ALT level between different groups (*F* (3, 28) = 5.5, *p* = 0.004). Serum ALT in the RIR group showed a significant increase compared to the control group (*p* < 0.05). Serum ALT in the RIR+TE and TE groups showed a significant decrease compared to the RIR group (*p* < 0.05). In this study, no significant difference was found between sham and TE groups in serum ALT (*p* > 0.05) (Figure 1(c)).

### 3.3. The Effect of TEO on Biochemical Parameters in the Liver following RIR

A significant statistical difference was observed between groups in liver MDA levels (*F* (3, 28) = 184.22, *p* < 0.0001). MDA levels in the RIR group showed a significant increase compared to the control group (*p* < 0.0001). MDA levels in the RIR+TE and TE groups showed a significant decrease compared to the RIR group (*p* < 0.0001). In this study, MDA level in TE group showed a significant increase compared to control group (*p* < 0.0001) (Table 3).

In this study, a significant difference was observed between the groups in liver NO levels (*F* (3, 28) = 116.72, *p* < 0.0001). NO levels in the RIR group showed a significant increase compared to the control group (*p* < 0.0001). NO levels in the RIR+TE and TE groups showed a significant decrease compared to the RIR group (*p* < 0.0001). NO level in the TE group showed a significant increase compared to control group (*p* < 0.0001) (Table 3).

In this study, a significant statistical difference in liver CAT activity was observed between different groups (*F* (3, 28) = 431.36, *p* < 0.0001). CAT activity in the RIR group showed a significant decrease compared to the control group (*p* < 0.0001). CAT activity in the RIR+TE group showed a significant increase compared to the RIR group (*p* < 0.001). CAT activity in the TE group showed a significant decrease compared to that in the control group (*p* < 0.0001) (Table 3).

In the present study, one-way ANOVA showed a significant difference between the groups in liver GPX activity (*F* (3, 28) = 183.95, *p* < 0.0001). Activity of GPX in the RIR group showed a significant decrease compared to the control group (*p* < 0.0001). Activity of GPX in the RIR+TE and TE groups showed a significant decrease compared to the RIR group (*p* < 0.0001). GPX activity in the TE group showed a significant decrease compared to the control group (*p* < 0.0001) (Table 3).

A significant statistical difference was observed between the groups in liver GSH levels (*F* (3, 28) = 84.68, *p* < 0.0001). GSH levels in the RIR group showed a significant decrease compared to the control group (*p* < 0.0001). GSH levels in the RIR+TE group showed a significant increase compared to the RIR group (*p* < 0.0001) and GSH levels in the TE group showed a significant decrease compared to the RIR group (*p* < 0.05). There was a significant decrease in GSH level in the TE group compared to control group (*p* < 0.0001) (Table 3).

### 3.4. The Effect of TEO on Gene Expression IL6, TNF-*α*, and Caspase-3 in the Liver following RIR

**Figure 2:**
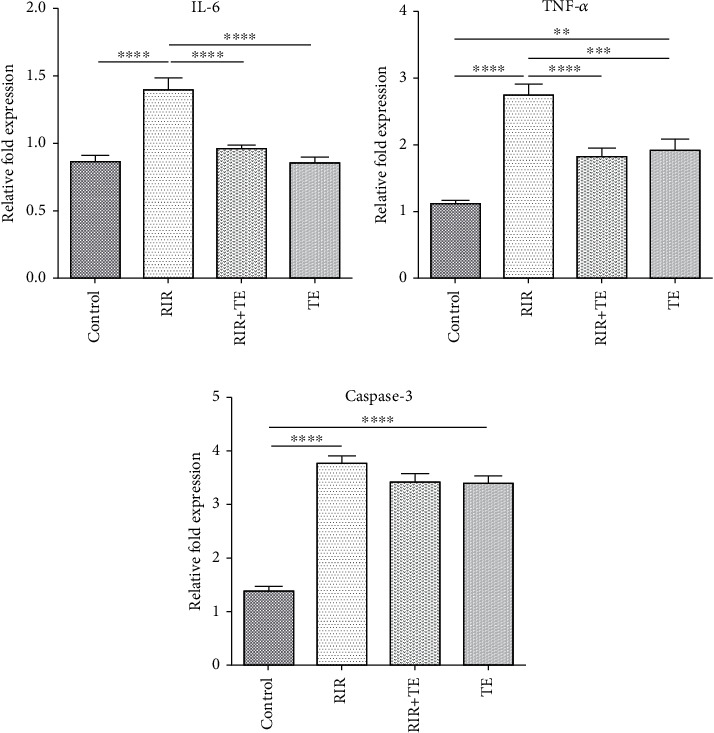
Effect of TE on the liver mRNA expression levels of inflammatory markers such as IL-6 and TNF-*α* and apoptotic marker such as caspase-3 following RIR. Data are expressed as mean + standard error mean (SEM). Values of this study were analyzed using one way ANOVA. Tukey test was used to compare the two groups. ^∗∗∗∗^ shows a significant difference between the two groups with *p* < 0.0001, ^∗∗∗^ shows a significant difference between the two groups with *p* < 0.001, ^∗∗^ shows a significant difference between the two groups with *p* < 0.01, and ^∗^ shows a significant difference between the two groups with *p* < 0.05.

In the present study, a significant difference in liver mRNA expression of IL-6 was observed between different groups (*F* (3, 28) = 20.58, *p* < 0.0001). The mRNA expression of IL-6 in the RIR group compared to the control group showed a significant increase (*p* < 0.0001). The mRNA expression of IL-6 in the RIR+TE and TE groups compared to that in the RIR group showed a significant decrease (*p* < 0.0001) (Figure 2(a)). A significant statistical difference was observed between the mRNA expression of TNF-*α* in the liver of different groups (*F* (3, 28) = 23.17, *p* < 0.0001). The mRNA expression of TNF-*α* in the RIR group compared to the control group showed a significant increase (*p* < 0.0001). The mRNA expression of TNF-*α* in the RIR+TE and TE groups compared to the RIR group showed a significant decrease (*p* < 0.05) (Figure 2(b)).

There was a significant difference between different groups regarding mRNA expression of caspase-3 in liver (*F* (3, 28) = 65.38, *p* < 0.0001). The mRNA expression of caspase-3 in the RIR group compared to the control group showed a significant increase (*p* < 0.0001). The mRNA expression of caspase-3 in the RIR+TE and TE did not show significant differences with the RIR group (*p* > 0.05). The mRNA expression of caspase-3 in the TE group showed a significant increase compared to that in the control group (*p* < 0.0001) (Figure 2(c)).

## 4. Discussion

Liver injury induced by RIR can cause damage to other organs and their death [20]. Currently, there is no suitable treatment for RIR-induced liver injury. However, improving liver function, restoring antioxidant levels, reducing inflammatory response, and reducing apoptosis can be useful in this case [20]. Previous studies found that antioxidants such as gallic acid, rosmarinic acid, and L-glutamine and selenium had the potential to reduce oxidative stress in the liver and kidney tissues and improve the function of these organs [21–25]. In our study, RIR increased the level of MDA, NO, and liver enzymes such as ALT, ALP, and AST, decreased activity of CAT and GPX, and decreased the level of GSH in liver tissue. Also, the expression of caspase-3 gene and inflammatory cytokines such as TNF-alpha and IL-6 increased in the liver. Gamma oryzanol reduced the oxidative stress caused by RIR in distant organs such as the brain, heart, and liver by increasing the antioxidant defense potential. This antioxidant reduced inflammation and apoptosis in the liver tissue and improved the function of this organ [26]. Also, during a study, the administration of naringenin reduced apoptosis, inflammation, and liver tissue damage caused by renal ischemia-reperfusion [27]. Berberine, with its antioxidant and anti-inflammatory activity, reduces liver dysfunction caused by renal ischemia-reperfusion in rats [28]. Alpha-lipoic acid as an antioxidant also reduced kidney damage in diabetic rats [29]. Therefore, antioxidants reduce organ damage caused by harmful factors. In the present study, thyme oil reduced liver enzymes, so it can improve liver function. A study by Yousefi et al. showed that fish fed a diet containing TE and immunogenic supplements had higher liver antioxidant parameters including CAT, glutathione reductase (GR), GPX, and superoxide dismutase (SOD) and lower MDA compared to the control group [30]. Also, in our study, thyme oil reduced the level of NO and MDA, so thyme oil can strengthen the antioxidant capacity and reduce the oxidative stress of liver tissue following RIR injury. Thyme extract reduces the production and expression of inflammatory mediators, TNF-*α*, IL-1*β*, and IL-6 [31]. In the present study, thyme oil with its anti-inflammatory properties was able to reduce the expression of proinflammatory cytokines such as IL-6 and TNF-*α* in the liver tissue. With reducing oxidative stress and inflammation and increasing antioxidant activity [32, 33], in the present study, liver function is improved by the reduction of serum ALT, AST, and ALP. Thyme extract improves dexamethasone-induced hepatotoxicity by reducing the activity of liver enzymes and by increasing the antioxidant activity of the liver [34]. TE, with its antioxidant properties, reduces oxidative and genetic damage caused by titanium toxicity [35]. According to previous studies, TE contains phenolic compounds, and after administration to rats with cadmium toxicity, it reduces lipid peroxidation and oxidative stress in liver tissue. Caryophyllene and linalool, as the main active ingredients in thyme, improve weight, reduce cadmium levels and inflammation, and improve antioxidant capacity in the liver [33]. Thyme essential oil containing carvacrol reduces renal and neurotoxicity induced by insecticide voliam targo [36]. Therefore, according to the previous studies and the results of our study, it can be said that thyme contains phenolic and antioxidant compounds that reduce inflammation and oxidative stress in different organs and thereby improve the function of that organ. Thymol and TE not only have antimicrobial, antifungal, and antiviral properties but also induce apoptosis in tumor cells [37]. Crocin decreases the apoptosis rate of diabetic rats through decreasing the expression of Bax and increasing the expression of Bcl2 [38]. Following the consumption of thyme oil, the expression of caspase-3 increased in the liver of rats. In a study conducted by Abdel-Wahhab et al., TE improved the antioxidant status and reduced apoptosis by decreasing the expression of Bax and increasing the expression of Bcl-2 in kidney and liver tissues [39]. In another study, by examining the Bcl2 gene, thymol and thyme oil were mentioned as having anti-inflammatory and antiapoptotic properties [40]. Carvacrol and thymol are the main components of thyme essential oil, which can produce reactive oxygen species and destroy tumors [41]. Carvacrol reduces pulmonary vascular remodeling under chronic hypoxia through inhibition of Bcl-2 expression and activation of caspase-3 [42]. 200 mg/kg thymol through anti-inflammatory, antioxidative, and antiapoptotic mechanisms attenuates indomethacin-induced gastric mucosal damage, but high-dose thymol (500 mg/kg) induces apoptosis through caspase-3 gene expression [43]. Thymol and carvacrol in high dose are likely to increase the apoptosis in tissues. Therefore, the possible reason for increase in apoptosis is high dose of TE and large amount of its components like thymol and carvacrol. However, this dose reduced inflammation and lipid peroxidation.

## 5. Conclusion

The results of our study showed that pretreatment with thyme essential oil for one week can protect the liver from damage caused by renal ischemia-reperfusion due to its antioxidant and anti-inflammatory effects. In this study, thyme oil showed apoptotic activity in liver tissue, in which it is suggested to investigate different doses of thyme oil in future studies.

## Figures and Tables

**Table 1:** Used primer sequences.

Gene	Primer sequence (5′→3′)	Length (bp)
*β*-Actin (F) *β*-Actin (R)	TATCGGCAATGAGCGGTTCC3 AGCACTGTGTTGGCATAGAGG3	150

Caspase-3 (F) Caspase-3 (R)	GGACAGCAGTTACAAAATGGATTA CGGCAGGCCTGAATGATGAAG	393

IL-6 (F) IL-6 (R)	CGAAAGTCAACTCCATCTGCC GGCAACTGGCTGGAAGTCTCT3	74

TNF-*α* (F) TNF-*α* (R)	CCAGGAGAAAGTCAGCCTCCT TCATACCAGGGCTTGAGCTC	87

**Table 2:** Effect of TE on initial body weight, final body weight, BMI, food intake, and water intake following RIR.

Group	Initial body weight (g)	Final body weight (g)	BMI (g/cm^2^)	Liver weight (g)	Food intake (g/rat/day)	Water intake (ml/rat/day)
Control	209.13 ± 3.7	213.75 ± 3.02	1.45 ± 0.05	5.19 ± 0.16^∗^	12.75 ± 0.59	53.63 ± 2.23
RIR	206.38 ± 5.04	207.88 ± 4.73	1.47 ± 0.02	5.79 ± 0.16^**$**^	12.85 ± 0.8	54.38 ± 2.19
RIR+TE	202.38 ± 4.39	208.5 ± 3.3	1.46 ± 0.04	5.25 ± 0.12^∗^	12.25 ± 0.86	53.13 ± 1.87
TE	204.63 ± 3.63	208.88 ± 2.76	1.43 ± 0.03	5.15 ± 0.11^∗^	11.87 ± 0.67	55.13 ± 2.75

Data are expressed as mean + standard error mean (SEM). Values of this study were analyzed using one way ANOVA. Tukey test was used to compare the two groups. Significance level of the data was considered *p* value < 0.05. ^∗∗∗∗^*p* < 0.0001 as compared with RIR, ^∗∗∗^*p* < 0.001 as compared with RIR, ^∗∗^*p* < 0.01 as compared with RIR, ^∗^*p* < 0.05 as compared with RIR, ^**$$$$**^*p* < 0.0001 as compared with control, ^**$$$**^*p* < 0.001 as compared with control, ^**$$**^*p* < 0.01 as compared with control, and ^**$**^*p* < 0.05 as compared with control.

**Table 3:** The effect of TE on liver biochemical parameters following RIR.

Group	MDA (*μ*mol/mg proteins)	NO (nmol/dl)	GSH (*μ*mol/mg proteins)	GPX (U/mg proteins)	CAT (U/mg proteins)
Control	72.3 ± 1.7^∗∗∗∗^	3.23 ± 0.09^∗∗∗∗^	4.98 ± 0.1^∗∗∗∗^	612.5 ± 9.18^∗∗∗∗^	60.37 ± 1.49^∗∗∗∗^
RIR	173.38 ± 3.22^**$$$$**^	4.94 ± 0.06^**$$$$**^	4.13 ± 0.03^**$$$$**^	517.88 ± 8.76^**$$$$**^	23.69 ± 0.24^**$$$$**^
RIR+TE	110 ± 4.6^∗∗∗∗^	4.17 ± 0.06^∗∗∗∗^	5.02 ± 0.04^∗∗∗∗^	459 ± 6.24^∗∗∗∗^	28.28 ± 0.57^∗∗^
TE	144.5 ± 2.6^∗∗∗∗^^**$$$$**^	3.73 ± 0.04^∗∗∗∗^^**$$$$**^	3.86 ± 0.05^∗^^**$$$$**^	367.13 ± 5.56^∗∗∗∗^^**$$$$**^	20.68 ± 0.71^**$$$$**^

Data are expressed as mean + standard error mean (SEM). Values of this study were analyzed using one way ANOVA. Tukey test was used to compare the two groups. Significance level of the data was considered *p* value < 0.05. ^∗∗∗∗^*p* < 0.0001 as compared with RIR, ^∗∗∗^*p* < 0.001 as compared with RIR, ^∗∗^*p* < 0.01 as compared with RIR, ^∗^*p* < 0.05 as compared with RIR, ^**$$$$**^*p* < 0.0001 as compared with control, ^**$$$**^*p* < 0.001 as compared with control, ^**$$**^*p* < 0.01 as compared with control, and ^**$**^*p* < 0.05 as compared with control.

## Data Availability

Upon a reasonable request, the data supporting the results of this article will be made available by the corresponding responsible author.
